# Supplementary material for: Role of lncRNA XIST/miR-146a Axis in Matrix Degradation and Apoptosis of Osteoarthritic Chondrocytes Through Regulation of MMP-13 and BCL2
Source: Biology (Basel). 2025 Feb 20;14(3):221. doi: 10.3390/biology14030221 (PMC11940272; doi:10.3390/biology14030221)
Supplement: Supplementary file 1 [file biology-14-00221-s001.zip › Figure S1.pdf]

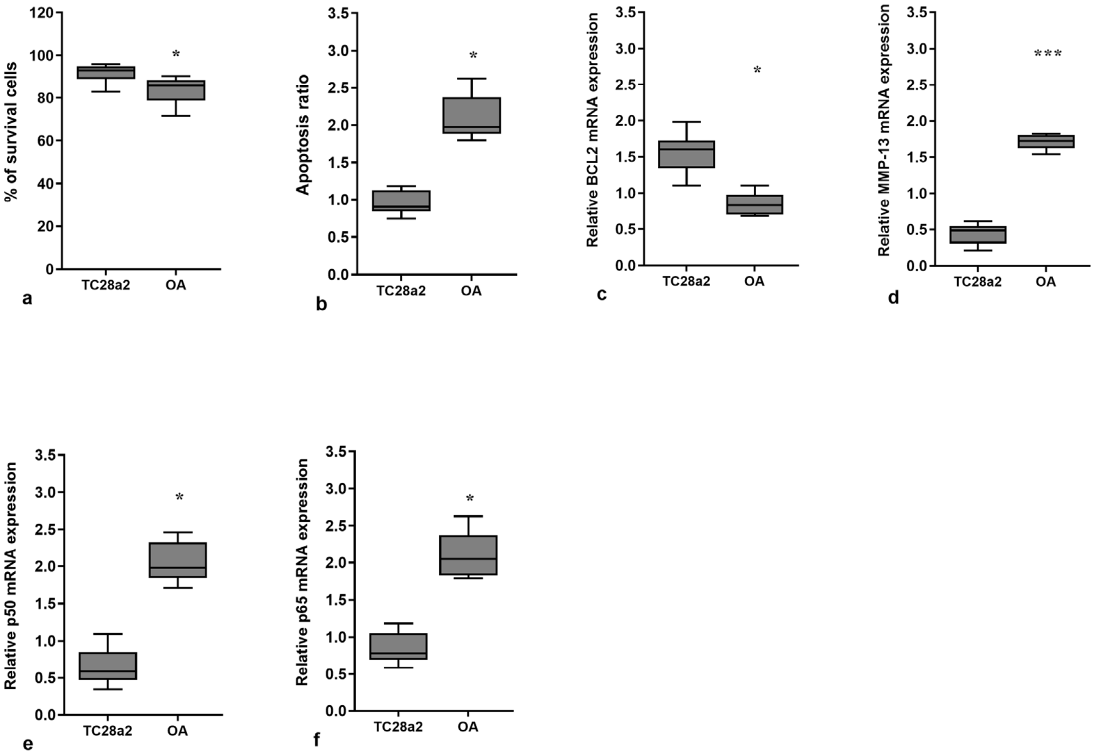

Figure S1. Differences in survival, apoptosis, and target genes expression between OA chondrocytes and normal cells T/C-28a2 at the basal condition. Legend: (**a**) Evaluation of cell viability by MTT assay, (**b**) Detection of apoptosis by flow cytometry, (**c**–**f**) gene expression of B-cell lymphoma (BCL2), metalloproteinase (MMP)-13, p65 and p50 subunits of nuclear factor (NF)-κB in T/C-28a2 cell line and OA chondrocytes assessed by quantitative real time PCR.
