# Supplementary material for: Role of lncRNA XIST/miR-146a Axis in Matrix Degradation and Apoptosis of Osteoarthritic Chondrocytes Through Regulation of MMP-13 and BCL2
Source: Biology (Basel). 2025 Feb 20;14(3):221. doi: 10.3390/biology14030221 (PMC11940272; doi:10.3390/biology14030221)
Supplement: Supplementary file 1 [file biology-14-00221-s001.zip › Table S1.pdf]

Table S1. List of primers used for quantitative real time PCR.

Genes	Cat. No. (Qiagen)
XIST	SI03654469
miR-146a	MS00003535
BCL2	QT00000721
MMP-13	QT00001764
NFKB1	QT00063791
RELA	QT01007370
SNORD-25	MS00014007
RNU6B_2	MS00014000
ACTB	QT00095431

Abbreviations: *XIST* = lncRNA X-inactive-specific transcript, *miR* = microRNA, *BCL2* = B-cell lymphoma 2, *MMP-13* = metalloproteinase 13, *NFKB1* = Nuclear Factor Kappa B Subunit 1 (p50), *RELA* = Proto-Oncogene, NF-KB Subunit (p65), *SNORD-25* = Small Nucleolar RNA, C/D Box 25, *RNU6B_2* = RNA, U6 Small Nuclear 2, *ACTB* = Actin Beta
